# Nitric oxide attenuated transforming growth factor-β induced myofibroblast differentiation of human keratocytes

**DOI:** 10.1038/s41598-021-87791-x

**Published:** 2021-04-14

**Authors:** Joo-Hee Park, Martha Kim, Bora Yim, Choul Yong Park

**Affiliations:** 1 Department of Biochemistry, College of Medicine, Dongguk University, Gyeongju, South Korea; 2 Department of Ophthalmology, Ilsan Hospital, Dongguk University, 814, Siksadong, Ilsan-dong-gu, Goyang, Gyunggido 410-773 South Korea

**Keywords:** Visual system, Drug development

## Abstract

Nitric oxide (NO) has the potential to modulate myofibroblast differentiation. In this study, we investigated the effect of exogenous NO on the myofibroblast differentiation of human keratocytes using sodium nitrite as a NO donor. Myofibroblasts were induced by exposing resting keratocytes to transforming growth factor (TGF)-β1. N-cadherin and α-smooth muscle actin (αSMA) were used as myofibroblast markers. Both resting keratocytes and -stimulated keratocytes were exposed to various concentrations of sodium nitrite (1 μM to 1000 mM) for 24 to 72 h. Exposure to sodium nitrite did not alter keratocytes’ viability up to a 10 mM concentration for 72 h. However, significant cytotoxicity was observed in higher concentrations of sodium nitrite (over 100 mM). The expression of αSMA and N-cadherin was significantly increased in keratocytes by TGF-β1 stimulation after 72 h incubation. The addition of sodium nitrite (1 mM) to TGF-β1-stimulated keratocytes significantly decreased αSMA and N cadherin expression. Smad3 phosphorylation decreased after sodium nitrite (1 mM) exposure in TGF-β1-stimulated keratocytes. The effect of NO was reversed when NO scavenger, 2-4-carboxyphenyl-4,4,5,5-tetramethylimidazoline-1-oxyl-3-oxide (cPTIO) was added in the culture medium. Application of sodium nitrite resulted in significant decrease of corneal opacity when measured at 2 weeks after the chemical burn in the mouse. These results verified the potential therapeutic effect of NO to decrease myofibroblast differentiation of human keratocytes and corneal opacity after injury.

## Introduction

Nitric oxide (NO) is a small signaling molecule with various biological functions. NO is endogenously produced by the activation of NO synthases (NOSs) under various conditions^1^. In pathologic states, NO acts as a free radical messenger and mediates inflammation and vasodilatation^1,2^. NO is also an important physiological regulator of cellular proliferation^3–5^. In a skin wound model, the exogenous NO supply facilitated the wound healing response^1,2^. In addition, NO deficiency leads to impaired wound healing, as shown in nitric oxide synthase (NOS) knock-out mice^6,7^. Furthermore, a potent anti-fibrotic action of exogenous NO was also reported^8^. NO activates soluble guanylate cyclase and increases the cyclic guanosine monophosphate (cGMP) level in cells^9^. The activation of the NO-guanylate cyclase-cGMP pathway was verified to attenuate fibrotic responses in organs, such as the liver, kidney, prostate, heart, skin, and lung^10–12^.

The results of previous studies suggest the potential therapeutic effect of NO in the healing process of corneal wounds. Recently, the application of exogenous NO in the ophthalmic field was actively investigated. The permissive role of NO in corneal epithelial wound healing was reported previously^13–17^. The topical application of NO successfully promoted the corneal epithelial wound healing process^13,17^. In addition, NO’s antibacterial effect is another benefit to prevent further corneal damage from bacterial infection after injury^18,19^. Incidentally, a recent development of NO as a promising anti-glaucoma medication further increased the clinical interest of NO in the ophthalmic field^9,20,21^.

Although NO ameliorates corneal epithelial wound healing, corneal injury usually results in both corneal epithelial and stromal damage simutaneously. Of course, keratocytes are the major cell component of corneal stroma. In corneal scars, keratocytes differentiate into myofibroblasts and lay down abnormal collagen fibers that can deteriorate corneal transparency. Therefore, the modulation of myofibroblast differentiation in an injured cornea is a critical therapeutic target to minimize corneal opacity and preserve clear vision. From this perspective, the evaluation of the effect of NO on keratocytes is a necessary step for the development of NO as a corneal wound healing modulator. Although the anti-fibrotic action of exogenous NO was reported in various human tissues, its role in corneal fibrosis, especially myofibroblasts’ differentiation from keratocytes, has not been fully elucidated. If NO is found to benefit both corneal epithelial cells and stromal cells, the further development of new drugs using NO can be more effective.

In this study, we investigated the effect of exogenous NO on primarily cultured human keratocytes. Different concentrations of NO donors (sodium nitrite) were applied in the culture media, and the cellular viability of keratocytes was measured. We induced keratocytes’ myofibroblast differentiation by adding transforming growth factor β1 (TGF-β1) to the culture media and investigated the effect of NO on myofibroblast markers’ expressions, N cadherin and α-smooth muscle actin (αSMA) from TGF-β1-stimulated keratocytes. Finally, the effect of NO on corneal opacity development was evaluated using murine chemical corneal burn model.

## Results

### Keratocytes’ viability with different concentrations of NO donors

**Figure 1:**
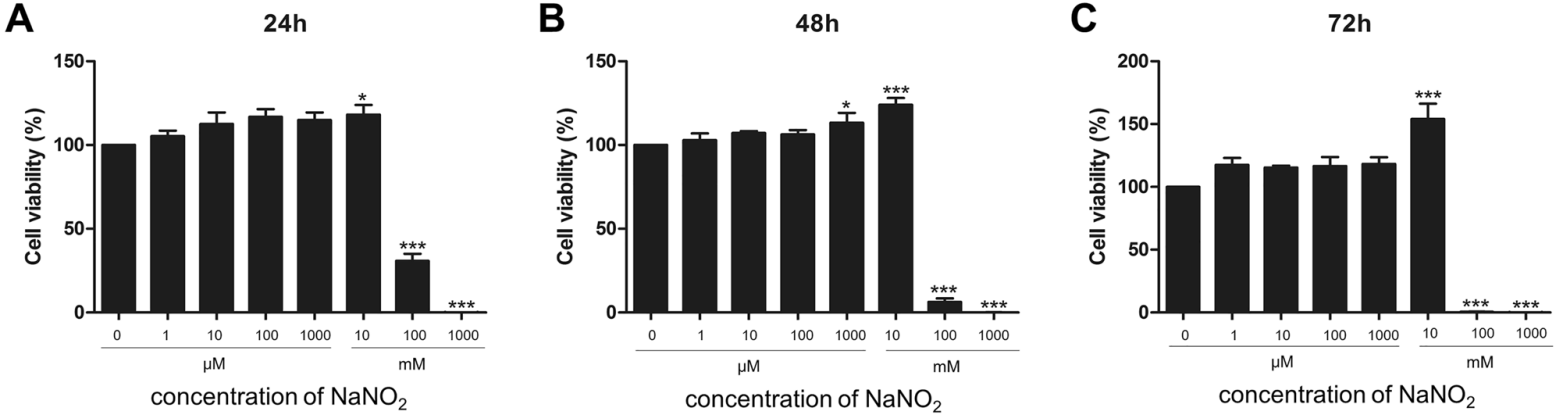
Keratocytes’ viability was measured after different concentrations (1 μM to 1000 mM) of sodium nitrite exposure for 24 (**A**), 48 (**B**) and 72 h (**C**). Sodium nitrite did not harm cultured keratocyte viability up to 10 mM concentration for 72 h. However, high concentrations (100 mM or 1000 mM) of sodium nitrite decreased keratocyte viability. *p < 0.05, ***p < 0.001.

**Figure 2:**
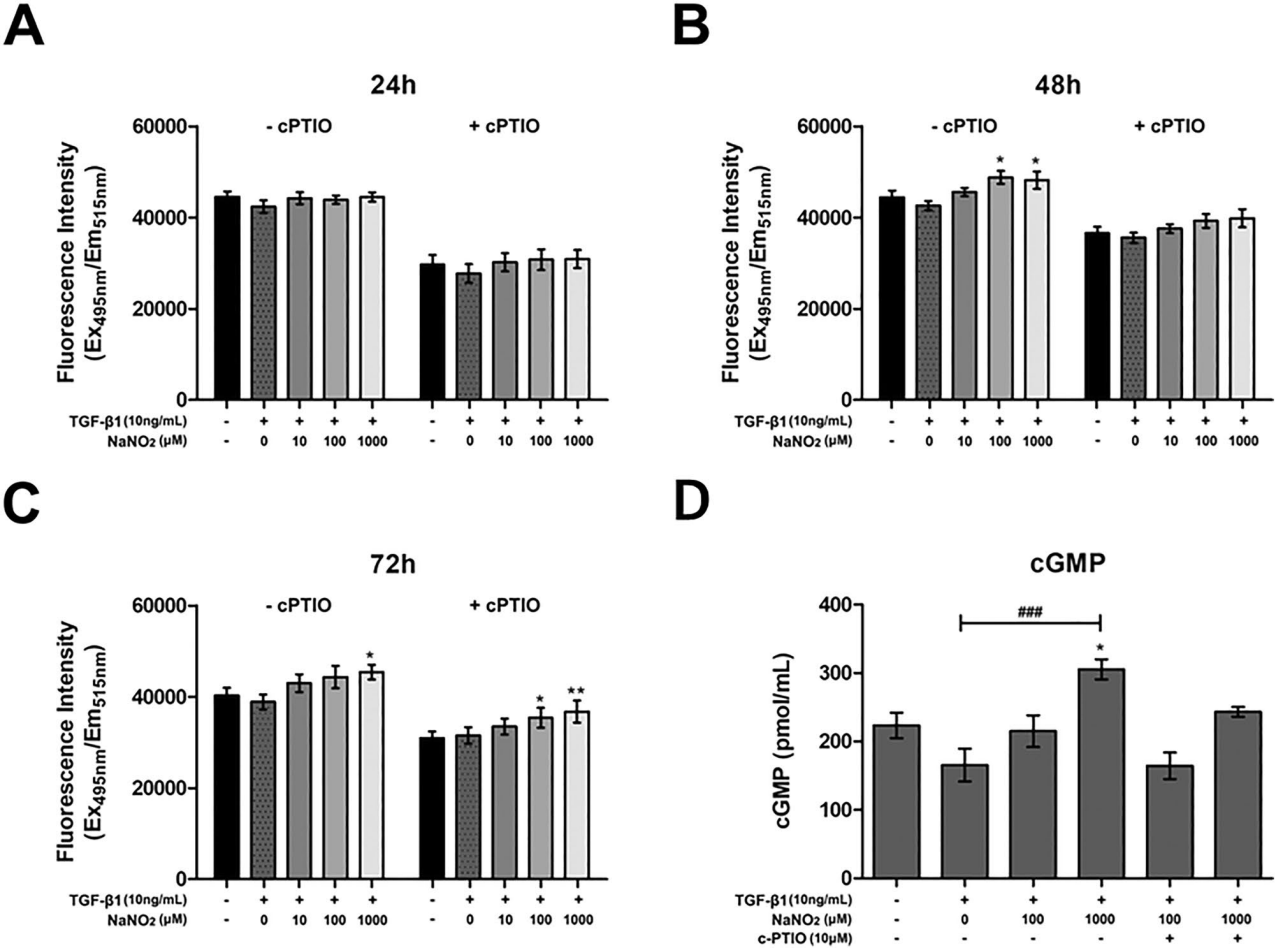
The effect of sodium nitrite on the intracellular NO and cyclic guanine monophosphate (cGMP) concentrations. (**A**–**C**) Sodium nitrite in the culture medium resulted in mild increase of intracellular NO concentration after 24 (**A**), 48 (**B**) and 72 h (**C**) incubation. Addition of 2-4-carboxyphenyl-4,4,5,5-tetramethylimidazoline-1-oxyl-3-oxide (cPTIO, 10 μM) in the culture medium scavenged NO and decreased intracellular NO concentration. (**D**) cGMP increased with the addition of sodium nitrite and addition of cPTIO blunted cGMP generation. *p < 0.05, **p < 0.01, ^###^p < 0.001.

We investigated any toxic effect of NO on keratocytes. Keratocyte viability was not deteriorated at low concentrations (up to 10 mM) of sodium nitrite. Rather mild increase of cell viability was observed with the addition of 10 mM of sodium nitrite. However, sodium nitrite decreased keratocytes’ viability at high concentrations (equal to or more than 100 mM). This toxicity increased with a longer incubation period. The decrease of viability in high concentrations (over 100 mM) of sodium nitrite is attributed to the hyper-osmolar stress induced by excess sodium in the culture media (Fig. 1). Intracellular NO concentration after exposure to different concentrations of sodium nitrite was measured (Fig. 2). A mild increase of intracellular NO concentration was observed after 24, 48 and 72 h incubation. Addition of 2-4-carboxyphenyl-4,4,5,5-tetramethylimidazoline-1-oxyl-3-oxide (cPTIO, 10 μM) in the culture medium scavenged NO and decreased intracellular NO concentration. NO activates guanylate cyclase and increase the production of cGMP. Significant increase of intracellular cGMP was observed after exposure to sodium nitrite. No significant change of oncogene activation related proteins, p53 and p21 was observed with the NO donor treatment keratocytes. (Supplement Fig. S1).

### mTOR pathway activation and cellular autophagy

**Figure 3:**
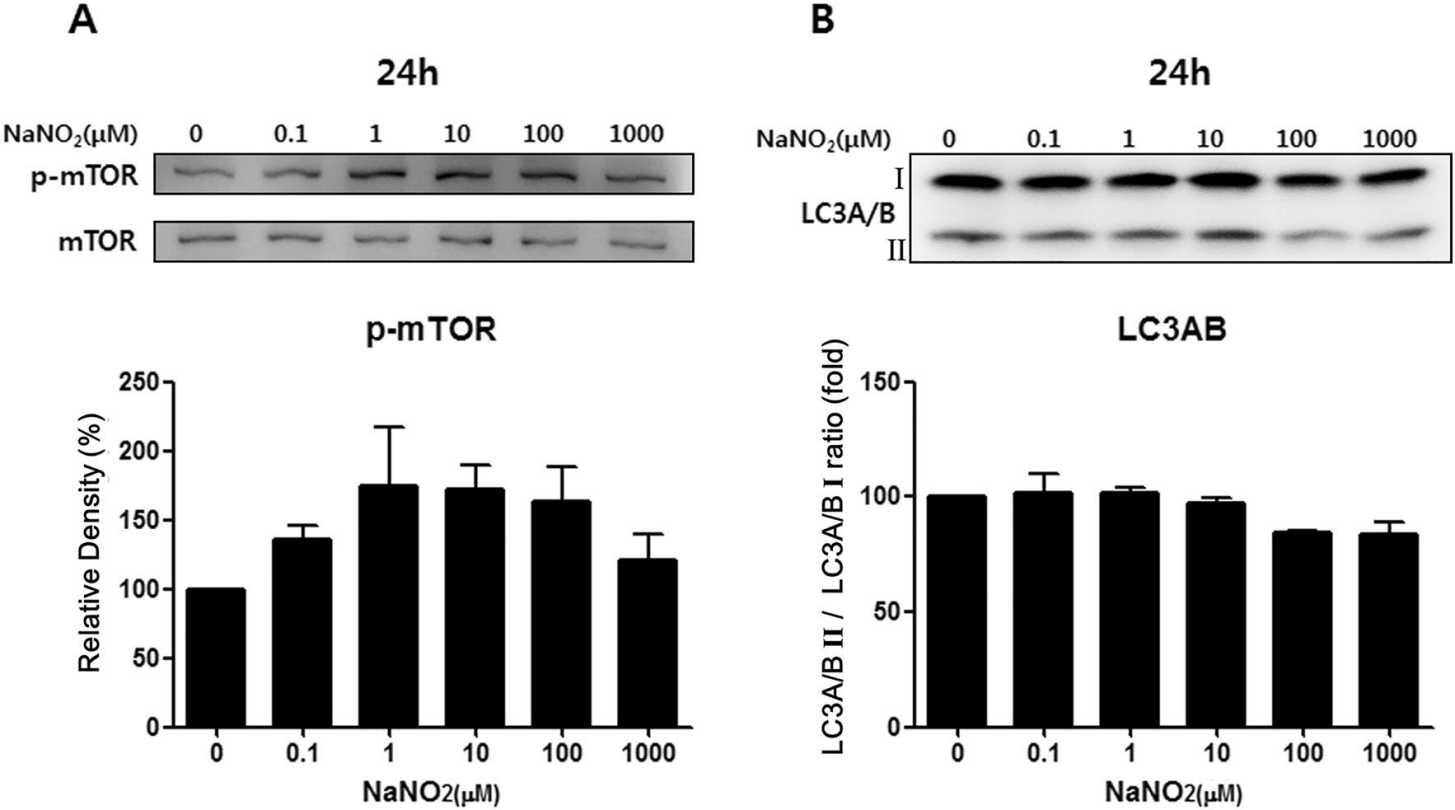
Sodium nitrite’s effect on two critical cellular survival pathways, mTOR and autophagy, was tested by Western blot. Phosphorylated mTOR is the activation of the mTOR pathway, while autophagy’s activation is represented by increases in LC3AB II forms. Sodium nitrite did not harm mTOR (**A**) and the autophagy pathway (**B**) of keratocytes up to 1 mM concentration for 24 h. Note that only the bands at the adequate molecular weights were shown here. Full length gel and blots are included in the Supplementary Information.

Two critical cell survival pathways (mTOR and autophagy) were not affected by sodium nitrite up to a 1000 μM concentration after 24-h exposure (Fig. 3).

### Keratocyte stimulation with TGFβ1

**Figure 4:**
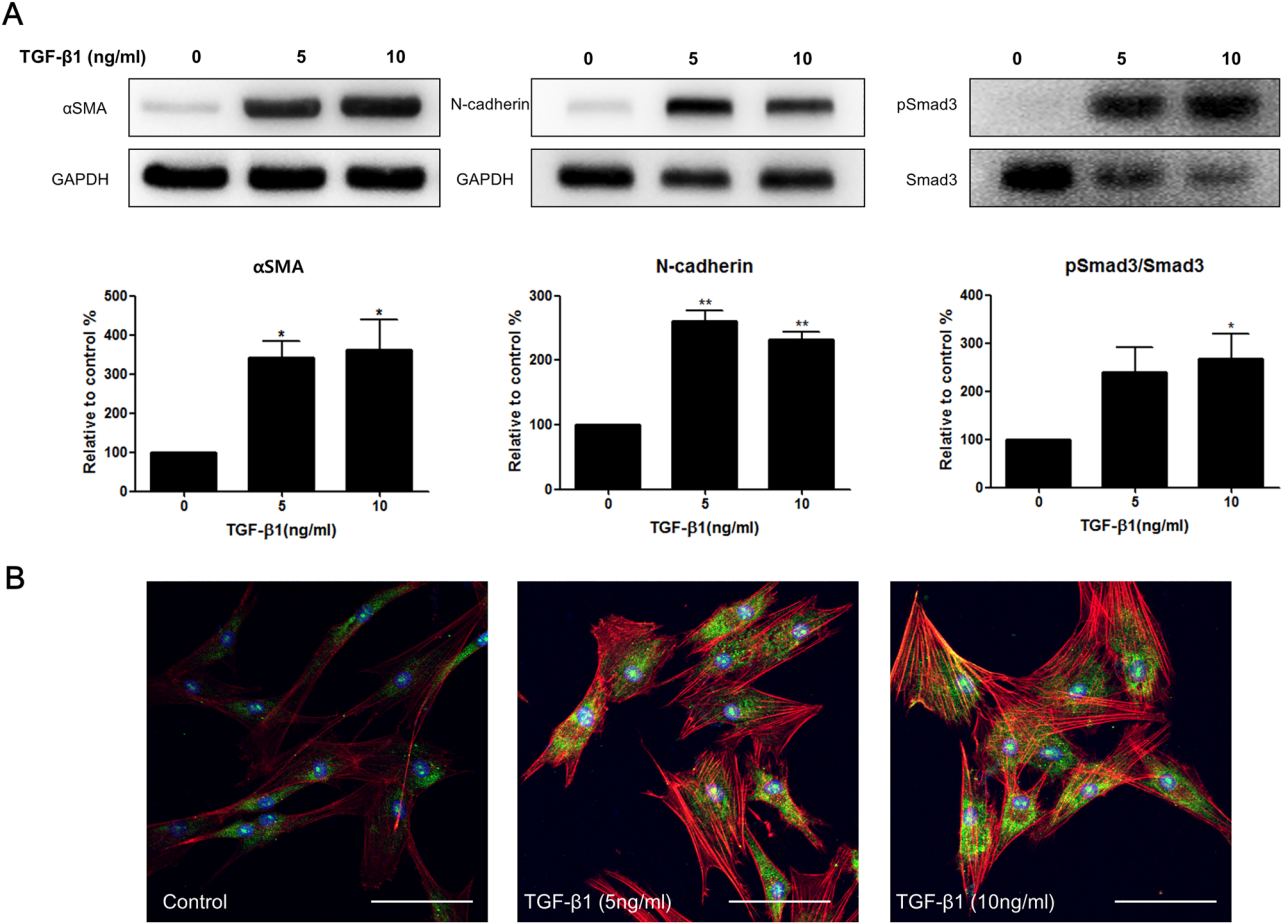
Myofibroblast marker expression by TGFβ1 stimulation in keratocytes. (**A**) Western blot analysis after 24 h TGFβ1 stimulation. TGFβ1 increased αSMA and N-cadherin expression from keratocytes. Smad3 phosphorylation is also increased with TGFβ1 stimulation. (**B**) Immunocytochemical staining revealed enhanced αSMA positivity (green: αSMA, red: F-actin, blue: DAPI, white bar: 50 μm) with higher concentrations of TGFβ1 stimulation for 72 h. Note that only the bands at the adequate molecular weights were shown here. Full length gel and blots are included in the Supplementary Information. *p < 0.05, **p < 0.01.

Myofibroblast differentiation from resting keratocyte after TGFβ1 stimulation for 24 h was evaluated by examining the expression level of pSmad3/Smad3, αSMA and N-cadherin. The phosphorylation of Smad 3 is an important pivotal step of the TGFβ1 pathway. Exposure of keratocytes to TGFβ1 significantly increased mlSmad3 phosphorylation (pSmad3) (Fig. 3A). Stimulation with TGFβ1 (2, 5, and 10 ng/) induced a dose-dependent increase of the αSMA expression in keratocytes (Fig. 4A,B). N-cadherin expression was also activated after TGFβ1 stimulation (Fig. 4A).

### ROS production in keratocyte stimulated with TGFβ1

**Figure 5:**
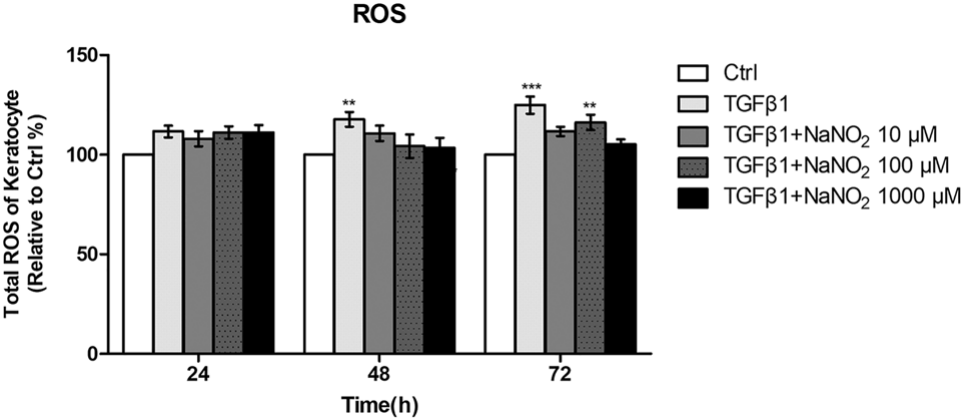
Total reactive oxygen species (ROS) production was measured in keratocytes after 24, 48 and 72 h culture with TGFβ1 (10 ng/mL). Slight increase of total ROS was observed in TGFβ1 (10 ng/mL) stimulated keratocytes compared to control keratocytes (Ctrl). Treatment with sodium nitrite (10, 100 or 1000 μM) alleviated total ROS production in stimulated keratocytes. **p < 0.01, ***p < 0.01.

Slight increase of total ROS was observed in TGFβ1 (10 ng/mL) stimulated keratocytes compared to control keratocytes. Treatment with sodium nitrite (10, 100 or 1000 μM) significantly alleviated total ROS production in TGFβ1 stimulated keratocytes (Fig. 5).

### Nitrite reductase activity in keratocyte

**Figure 6:**
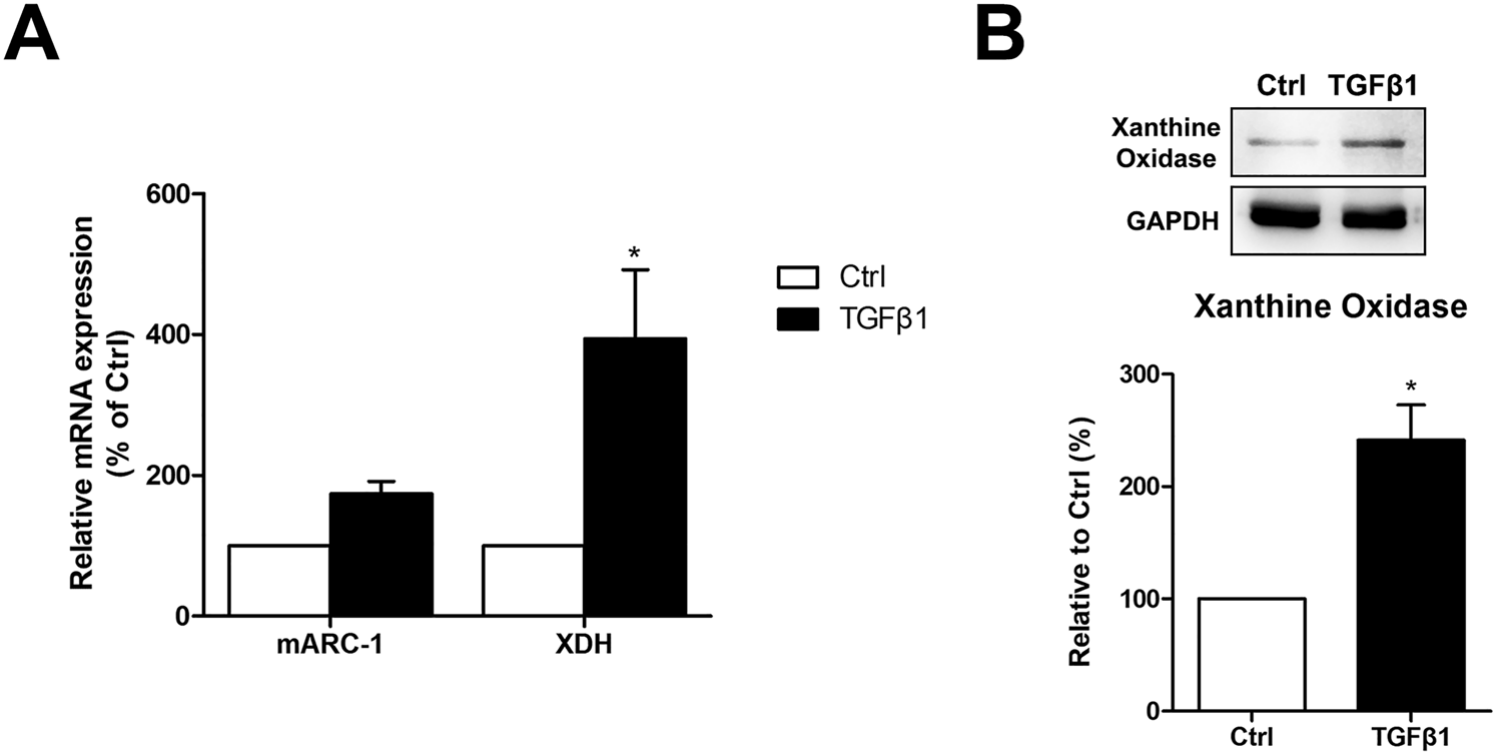
Nitrite reductase activity in keratocyte was measured in keratocytes using mitochondrial amidoxime reducing component 1 (mARC1), xanthine dehydrogenase (XDH) and xanthine oxidase. The mRNA expression level of mARC1 and xanthine dehydrogenase was measured with quantitative polymerase chain reaction while the protein level of xanthine oxidase was measured with western blot. TGFβ1 (10 ng/mL) stimulation increased xanthine dehydrogenase gene expression (**A**) and xanthine oxidase protein level (**B**) significantly in keratocytes. *p < 0.05.

Nitrite reductase activity in keratocyte was measured in keratocytes using mitochondrial amidoxime reducing component 1 (mARC1), xanthine dehydrogenase and xanthine oxidase. The mRNA expression level of xanthine dehydrogenase was significantly increased after TGFβ1 stimulation while the mRNA expression level of mARC1 showed little change. The protein level of xanthine oxidase was also increased after TGFβ1 stimulation (Fig. 6).

### NO’s effect on αSMA expression from TGFβ1-stimulated keratocytes

**Figure 7:**
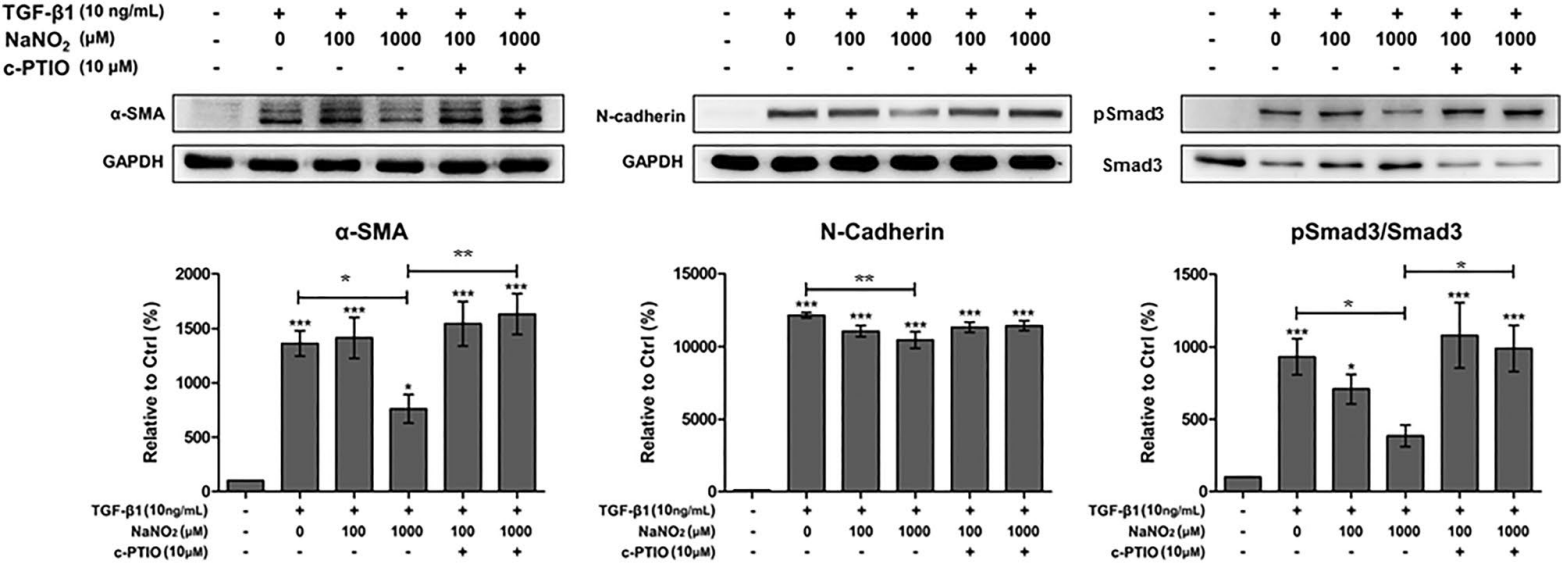
The effect of NO on TGFβ1-stimulated keratocytes. αSMA and N-cadherin expression in TGFβ1-stimulated keratocytes significantly decreased after 1000 μM sodium nitrite treatment for 24 h. However, lower concentration (100 μM) of sodium nitite showed little effect. Smad3 phosphorylation was also decreased after 1000 μM sodium nitrite treatment for 24 h. Addition of 2-4-carboxyphenyl-4,4,5,5-tetramethylimidazoline-1-oxyl-3-oxide (cPTIO, 10 μM) in the culture medium reversed the effect of NO. Statistical analysis was performed by setting the expression level in TGFβ1-stimulated keratocytes with no NO treatment as the baseline. Note that only the bands at the adequate molecular weights were shown here. Full length gel and blots are included in the supplementary information. *p < 0.05, **p < 0.01, ***p < 0.01.

**Figure 8:**
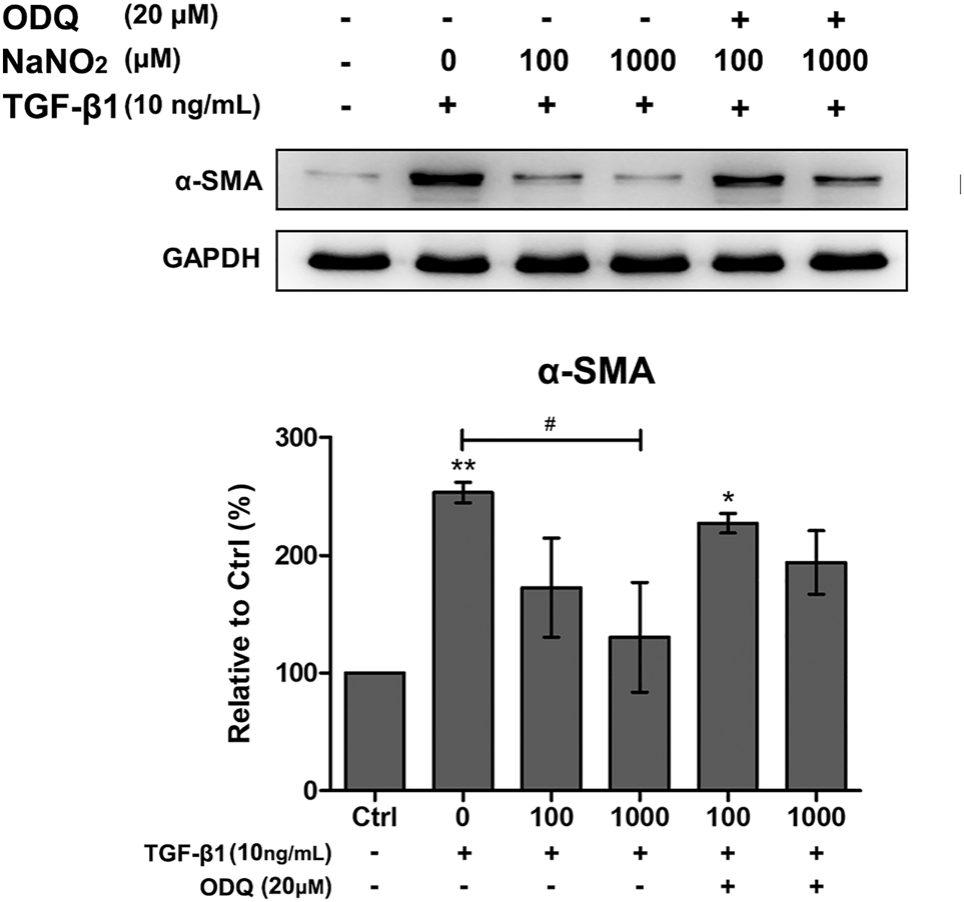
Evaluation of cGMP dependent pathway using guanylate cyclase inhibitor, ODQ (20 μM), in TGFβ1 (10 ng/mL) stimulated keratocyted. Addition of ODQ eliminated the anti-myofibroblastic effect of NO donors (DETA NONOates and sodium nitrite) as demonstrated by the restored α-SMA expression*.* Statistical significance was determined using one-way ANOVA followed by the Bonferroni multiple comparison test and the significant difference compared to no treated control (*p < 0.05, **p < 0.01); ^#^significant difference compared to TGFβ1 treated group (^#^p < 0.05).

We evaluated the effect of sodium nitrite treatment on TGFβ1-stimulated keratocytes. We added various concentrations (100 and 1000 μM) of sodium nitrite into the culture media. The addition of 1000 μM of sodium nitrite significantly decreased Smad3 phosphorylation, N-cadherin and αSMA expression in TGFβ1-stimulated keratocytes. However, lower concentrations (100 μM) of sodium nitrite failed to show any significant effect (Fig. 7). Addition cPTIO (10 μM) in the culture medium scavenged NO effect. The effect of sodium nitrite on αSMA expression in TGFβ1-stimulated keratocytes was further verified by using a different NO donor, DETA NONOate (Supplement Fig. S2). Inhibition of soluble guanylate cyclase by ODQ successfully reversed αSMA expression in TGFβ1-stimulated keratocytes (Fig. 8). This finding suggests that the role of cGMP is critical in NO mediated prevention of myofibroblast differentiation from keratocytes.

### In vivo effect of NO on corneal opacity development after injury

**Figure 9:**
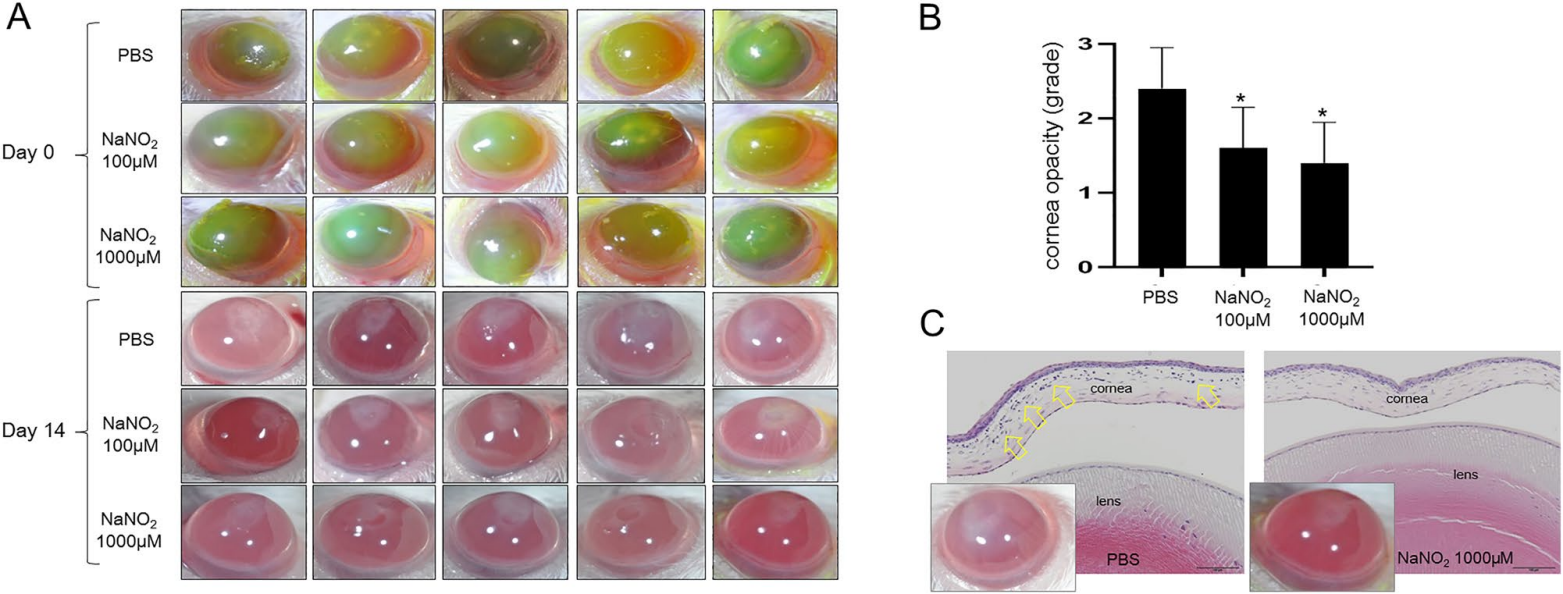
In vivo effect of NO on corneal opacity development after alkali burn. (**A**) Representative photographs of the alkali burned murine cornea at day 0 and 14 days after injury. Green stained areas by fluorescein at day 0 represent corneal epithelial defect induced by alkali burn. Corneal opacity of NO treated corneas (100 μM and 1000 μM) was significantly better than PBS control observed at 2 weeks. (**B**) The difference of corneal opacity grades between treatments and control (PBS) was significant. However, the difference between 100 and 1000 μM of sodium nitrite treatments was not significant. (**C**) Histologic examination revealed significantly increased corneal thickness and stromal cellularity (yellow arrows) in PBS treated cornea compared to NO treated cornea. *p < 0.05, scale bar: 100 μm.

Topical treatment with sodium nitrite (100 μM and 1000 μM) significantly decreased the corneal opacity development after alkali burn. The effect of both concentrations of sodium nitrite was similar. Histologic examination of revealed decreased corneal edema and cellularity (representing residual corneal inflammation) in corneal stroma in NO treated eyes compared to PBS control (Fig. 9).

## Discussion

In this study, we investigated the effect of NO on myofibroblast differentiation from human keratocytes. The safety of NO with keratocytes was verified by the maintenance of cell viability and intact mTOR/autophagy pathways observed up to 1 mM of sodium nitrite. The addition of 1 mM of sodium nitrite in the culture media resulted in significant decrease of the intracellular cGMP level, expression of αSMA and N-cadherin from TGFβ1-stimulated human keratocytes. Blocking the cGMP formation by soluble guanylate cyclase inhibitor (ODQ) reversed the NO effect on αSMA expression. Application of topical NO in the setting of chemical corneal burn resulted in significantly decreased corneal opacity.

Transforming growth factor-β1 (TGF-β1) is a strong inducer of myofibroblast differentiation through a pro-oxidant shift in redox homeostasis, which is associated with decreased NO/cGMP signaling^10^. ROS derived from NADPH oxidase (NOX4) mediated αSMA and collagen production by intestinal or nasal myofibroblasts when stimulated with TGF β^22,23^. After corneal injury, it is known that TGF-β1 produced in corneal epithelial cells can leak through the break of the epithelial basement membrane and activate keratocytes into myofibroblasts^24^. Therefore modifying NO/cGMP signaling pathway can be a potential therapeutic target to minimize corneal opacity in pathologic condition. It is reported that decreases of cGMP level at the wounding site can drive the myofibroblast differentiation of dermal fibroblasts. Conversely, the combinatorial effect of activators of soluble guanylate cyclase and inhibitors of cGMP degradation may lead to an elevation of cGMP signaling and induce the reversal of myofibroblast differentiation, as demonstrated in prostatic and dermal stromal cells^10^. As mentioned earlier, NO activates soluble guanylate cyclase and increases cGMP level in cells^9^. Therefore, the result of our current study is in line with previous reports about inhibitory NO effect on myofibroblast differentiation. In addition, as shown in Fig. 5, the increase of ROS can be alleviated by NO treatment.

Myofibroblasts play important roles in the corneal wound healing process^24–26^. Immediately after corneal injury, various cytokines, growth factors, and chemokines orchestrate the corneal wound healing process^26^. During the acute phase of corneal injury, damaged corneal epithelial cells produce profuse pro-inflammatory cytokines, such as interleukin 1 (IL-1), TGF-β1, and platelet-derived growth factor (PDGF), and these cytokines induce apoptosis of keratocytes at the injured area^27^. After the acute phase, keratocytes from the adjacent corneal stroma start to proliferate and migrate into the injured area. TGF-β1 can generate myofibroblasts from activated keratocytes^24^. The keratocyte derived myofibroblasts express several intermediate filament proteins, such as αSMA, vimentin, and desmin, which are important for providing mechanical strength to the injured tissue^28^. In addition, disorganized collagen fibers are produced by activated myofibroblasts. These abnormal collagen fibers and their irregular arrangement are the major causes of corneal opacity after wound healing.

Our finding that NO could decrease αSMA expression from activated keratocytes has an important clinical relevance, because the increased expression of αSMA is considered the key step toward myofibroblast differentiation. We tested various concentrations of NO donor, sodium nitrite, in the current study, because NO’s biologic effect is known to be dependent on its concentration. Previously, lower concentrations of NO were reported to exert a direct positive effect on various cellular proliferations, whereas higher concentrations of NO may have cytotoxic effects possibly through both oxidation and nitrosative stresses^3,29^.

We found that exogenous NO could prevent myofibroblasts differentiation with little harmful effect on keratocyte’s viability. This finding is an important clue that NO releasing treatment platforms can be safely used in various corneal traumatic or infectious diseases. We previously reported that exogenous NO can facilitate cornea epithelial cell healing after the mechanical injury^30^. Haze control after photorefractive keratectomy can be another implication of exogenous NO treatment because activation of stromal myofibroblasts was known as the main cause of delayed corneal haze after PRK^25^. Although mitomycin C (MMC) is wildly used to prevent corneal haze in the clinical setting, the long term safety issue of MMC has yet to be verified^31,32^. An ideal therapeutic agent to prevent post PRK corneal haze should be safe for keratocytes and can specifically block myofibroblast differentiation. In this respect, NO can be a promising candidate when considering its safety and efficacy.

Sodium nitrite not only stimulates cGMP production, but also promotes S-nitrosylation of cellular proteins by forming RSNO^33,34^. As shown in our study (Supplement Fig. S3), addition of sodium nitrite in TGFβ1-stimulated keratocytes significantly increased RSNO production, which can increase S-nitrosylation of corneal proteins. Investigation of protein functional change by NO induced S-nitrosylation in cornea can be an interesting topic for the future study.

Our study has some limitations. The first is the relatively short-term effect of NO on keratocytes that we observed. Because corneal opacity usually develops 2 to 4 weeks after injury in human, our in vitro data cannot provide the long-term effect of NO on the myofibroblast differentiation of keratocytes and its maintenance. Although chemical burn model was produced in a mouse model, it is possible that the wound healing response of mouse can be different from human. Therefore, the result is not always repeatable in human because of the many confounding factors of the human ocular surface. Another limitation is that sodium nitrite was used as an NO donor. Sodium nitrite is one of the widely accepted NO donors in various experimental settings. However, NO’s release from sodium nitrite necessitates nitrite reductase. Therefore, the final NO concentration after sodium nitrite exposure largely depends on cellular nitrite reductase levels. Although we verified sodium nitrite reductase activity in resting keratocytes and its increase with TGFβ1 stimulation, accurate titration of NO supply was impossible with the current setting of experiment. For a more accurate titration of the NO supply, it is more desirable to develop enzyme-independent NO delivering platforms in the future.

In conclusion, we found exogenous NO prevented myofibroblastic differentiation from TGF-β1-stimulated human keratocytes. These findings suggest the future use of exogenous NO-releasing drug platforms for the treatment of various ocular diseases threatening corneal transparency.

## Materials and methods

### Human keratocytes’ culture

The primary culture of human keratocytes was performed performed using a cadaveric donor corneal tissue not suitable for clinical use (from Eversight Korea, Seoul, South Korea) as described earlier^35^. Informed consent was obtained from next of kin of the cadaver donor for the tissue to be used in research. The use of human keratocytes was approved by the Institutional Review Board of Dongguk University Ilsan Hospital (IRB No: 2019-03-001) and the research was performed in accordance with the Declaration of Helsinki. Briefly, Descemet’s membrane and epithelium were gently removed using forceps and an ophthalmic knife from the donor corneal button. The corneal stroma was minced in a laminar flow hood. Subsequently, mid-stroma and posterior stroma explants were suspended in a culture medium and cultured in 24-well plates. The corneal stroma was sliced into quarters and digested overnight with 2.0 mg/mL collagenase (Roche Applied Science, Mannheim, Germany) and 0.5 mg/mL hyaluronidase (Worthington Biochemicals, Lakewood, NJ, USA) in DMEM at 37 °C. Isolated cells were washed in DMEM and cultured in Keratinocyte SFM (Gibco BRL, Carlsbad, CA, USA). The cells were cultured on tissue culture-treated plastic at 4 × 10^4^ cells/cm^2^. After reaching confluency, cells were harvested and suspended in a culture medium. The cells were plated in 75 cm^2^ tissue flasks and maintained at 37 °C in 5% CO_2_ and 95% air. The culture medium was changed every three days, and the cells were passaged using 0.25% Trypsin–EDTA (Gibco BRL, Carlsbad, CA, USA). Passage numbers 5–7 were used in this study.

### Cell viability assay

Cell viability assays were performed using a cell counting kit reagent (CCK-8; Dojindo Molecular Technologies, Inc., Kumamoto, Japan) according to the manufacturer’s protocol^35^. Briefly, keratocytes were cultured at 3 × 10^3^ cells per well in a 96-well plate and incubated for 24 h. Following the adherence of cells, different concentrations of sodium nitrite were added to the culture media for 24 h, 48 h, and 72 h. The wells with no sodium nitrite and the wells with a dimethyl sulfoxide (DMSO) addition were used as negative and positive controls, respectively. After the appropriate incubation, 10 uL of CCK-8 solution was added to each cultured well, and the absorbance was measured at 450 nm after 2 h incubation of keratocytes with the reagent. Cell viability in various sodium nitrite solutions was represented as a relative percentage compared to the negative control.

### TGFβ1 stimulation and sodium nitrite treatment

Keratocytes were stimulated by TGFβ1 (catalog number T7039, Sigma-Aldrich Corp., St. Louis, MO, USA) (5 or 10 ng/mL) for 24 h. The expression of αSMA, N-cadherin, Smad3 and phosphorylated Smad 3 were evaluated by Western blot. For sodium nitrite treatment, various concentrations (0, 10, 100, 1000 μM) of sodium nitrite were added to the culture media after 24 h of TGFβ1 (10 ng/mL) stimulation. After additional 24 h of incubation with sodium nitrite, cells were harvested for further analysis. For DETA NONOate treatment, 10 and 100 μM of DETA NONOates (catalog number ALX-430-014, Enzo Life Science, Lausen, Switzerland) were added to the culture media after 24 h of TGFβ1 (10 ng/mL) stimulation. The expression level of αSMA was evaluated by Western blot.

### Intracellular NO detection

Keratocytes were seeded at a density of 3 × 10^3^ cells per 1 well and grown on 96well black plates. And cells were treated 10 ng/mL of TGFβ1 and sodium nitrite (0, 10, 100, 1000 μM). NO quenching was done with 10 µM of 2-4-carboxyphenyl-4,4,5,5-tetramethylimidazoline-1-oxyl-3-oxide (cPTIO, catalog number C221, Sigma-Aldrich Corp.). After 24, 48, and 72 h of incubation, 5 µM of 4-amino-5-methylamino-2′,7′-difluoroscein (DAF-FM; catalog number D-23841, Invitrogen, Eugene, OR, USA) was added for 30 min at 37 °C. Cells were washed with DPBS, three times, and were incubated for an additional 20 min. Finally the fluorescence of each well plate was measured with excitation and emission at 495 and 515 nm, respectively.

### Reactive oxygen species (ROS) assay

Total ROS was detected using DCFDA/H2DCFDA-Cellular ROS Assay Kit (Cat. No. ab113851: Abcam, Cambridge, UK). Following treatment of TGFβ1 (10 ng/mL) or NaNO_2_ (10, 100, 1000 μM), cellular ROS was measured at 24 h, 48, and 72 h time point. As mentioned in manufacturer’s protocol, the cells were stained with 20 µM of 2′,7′-dichlorofluorescin diacetate (DCFDA) solution and incubated at 37 °C in the dark condition for 45 min. After discarding the solution, 100 µL/well of 1× buffer was added. The fluorescence was immediately measured at 485 nm excitation/535 nm emission.

### Measurement of total s-nitrosothiol

The measurement of total S-nitrosothiols (RSNO) was carried out using Griess/Saville method. Briefly, TGFβ1 (10 ng/mL) stimulated keratocytes were cultured for 24 h with or without sodium nitrite (0, 100, 1000 μM). The cell lysates were mixed with equal volumes of 1× Griess reagent (catalog number: G4410, Sigma Aldrich) which was freshly prepared with or without 3 mM HgCl_2_. The absorbance was measured at 540 nm and the amount of RSNO was calculated using a standard curve.

### Cyclic guanosine monophosphate (cGMP) assay

The intracellular cGMP level was measured using cGMP complete ELISA kit (catalog number ADI-900-164, Enzo Life Science, Farmingdale, NY, USA). According to the manufacturer’s protocol, briefly, 1 × 10^6^ cells were prepared for each sample and resuspended in 0.1 M-HCl. After incubaton for 10 min on ice, cell lysates were centrifuged and transferred to the assay plate. Following attachment of cGMP antibody and conjugate, assay plate was measured at the absorbance at 405 nm. Further evaluation of cGMP dependent pathway was performed using a soluble guanylate cyclase (sGC), 1H-[1,2,4] Oxadiazolo [4,3-a] quinoxalin-1-one (ODQ; catalog number o3636, sigma aldrich). After 30 min preincubation with ODQ (20 μM), keratocytes was treated with TGFβ1 (10 ng/mL) and sodium nitrite (100 or 1000 μM) for 24 h. The expression level of αSMA was evaluated by Western blot.

### Western blot analysis

The procedure for western blot was performed following the previously reported protocols^28^. Keratocytes were lysed in ice-cold radio immunoprecipitation assay (RIPA) buffer (50 mM Tris–HCl (pH 8.0), 150 mM NaCl, 1% NP-40, 0.5% deoxycholate, and 0.1% SDS) for 30 min. The debris was removed by centrifugation at 16,000×*g* for 1 min. Equal amounts (20 μg) of total cell protein were separated by SDS–polyacrylamide gel electrophoresis (SDS-PAGE) and transferred to the polyvinylidene difluoride (PVDF) membrane. After blocking with 5% BSA in TTBS buffer (10 mM Tris, pH 8.0, 150 mM NaCl, 0.1% Tween 20) for 1 h at room temperature, membranes were incubated overnight at 4 °C with the following primary antibodies: mouse anti-α smooth muscle actin (1:1000; catalog number: ab7817; abcam, Cambridge, UK), mouse anti-N-cadherin (1:1000; catalog number: 33-3900, thermo fisher scienetific, USA) rabbit anti-smad3 (1:1000; catalog number: ab40854; abcam), rabbit anti-phosphorylated smad3 (phosphor S423 + S425) (1:1000; catalog number: ab52903; abcam), mouse anti-p53 (1:1000; catalog number: sc-126; Santa Cruz) and mouse anti-p21 (1:1000; MAB1047, R&D Systems, Inc. Minneapolis, MN, USA), xanthine oxidase antibody (1:1000; catalog number: ab133268; abcam) and mouse anti-GAPDH (1:1000; catalog number: sc-365062; Santa Cruz, Biotechnology, Dallas, TX, USA). The membranes were incubated with peroxidase-conjugated secondary antibody for 1 h at room temperature. Blots were developed using an enhanced chemiluminescence kit (catalog number: RPN2232; GE Healthcare, Buckinghamshire, UK) and visualized using a Fujifilm Image Reader LAS-3000 (Fujifilm, Tokyo, Japan). Each experiment was repeated at least three times, and the densitometric analysis was performed using the Multi Gauge V3.0 (Fujifilm Life Science, Tokyo, Japan).

### Real time quantitative polymerase chain reaction (qPCR)

Total RNA was extracted from normal keratocyte (Ctrl) and TGFβ1 (10 ng/mL) stimulated keratocyte following TRiZol (Invitrogen) method. DEPC-water reconstituted RNA was used as template of cDNA, reverse transcriptase PCR was carried out by SuperScript III first strand synthesis system (Invitrogen) according to the manufacturer's instructions. The final products were used as a template on LightCycler 480 (Roche, Mannheim, Germany), using the SYBR Green I master (Roche). The primer sequences used in this study are mitochondrial amidoxime reducing component 1 (forward: 5′-CAC AGT GGG GAG TCA CAA AC-3′, reverse: 5′-AGG GAG AAG GAG AGG AGG AG-3′), xanthine dehydrogenase (forward: 5′-GAC TGT CAA TTC CCC TTC CT-3′, reverse: 5′-CTC TTT ACC AAC CGC AGA AA-3′) and GAPDH (forward: 5′-GTC TCC TCT GAC TTC AAC AGC G-3′, reverse: 5′-ACC ACC CTG TTG CTG TAG CCA A-3′).

### Immunocytochemistry

The procedure for immunocytochemistry was performed following the previously reported protocols^28^. Keratocytes were seeded at a density of 2 × 10^4^ cells per milliliter and grown on 4-well Lab-Tek chamber slides (Nalgene Nunc International, Penfield, NY, USA), and 0, 5, and 10 ng/mL of TGF-β1 were treated for 72 h. Cells were fixed with 3.7% paraformaldehyde for 10 min at room temperature, and permeabilization was conducted using 0.1% triton x-100 for 5 min at RT. Following the washing steps with DPBS, cells were blocked using 1% bovine serum albumin (BSA) in DPBS for 30 min at room temperature. The chamber slides were incubated overnight at 4 °C with rabbit polyclonal anti-α smooth muscle actin (1:1000; catalog number: sc-53142; Santa Cruz, Biotechnology). The chamber slides were then washed with DPBS and incubated with Alexa488-conjugated donkey anti-mouse antibody (1:1000; catalog number: A21202; Molecular Probes) for 2 h at room temperature. Staining for F-actin was executed using tetramethylrhodamine isothiocyanate (TRITC)-conjugated phalloidin (1 µg/mL; Sigma-Aldrich, St. Louis, MO, USA). The counterstaining of cell nuclei was carried out using 4′,6-diamidino-2′-phenylindole (DAPI, 10236276001; Roche Diagnostics GmbH, Mannheim, Germany) with mounting solution. Slides were viewed using a fluorescence microscope.

### Animal experiment

To investigate the in vivo effect of NO on corneal wound healing, Balb/c mice (15, males) were used. Animals were treated in compliance with the ARVO Statement for the Use of Animals in Ophthalmic and Vision Research and the ARRIVE (Animal Research Reporting of In Vivo Experiments) guidelines. All experimental protocol was approved by the Institutional Animal Care and Use Committee (IACUC) of Dongguk University, Ilsan Hospital (reference number: 2016-03146). Alkali burn was induced on right corneas of the mice as previously described^28^. Briefly, after topical anesthesia with 0.5% proparacaine hydrochloride (Alcaine, Alcon laboratory, Fort Worth, TX), a 0.1 N NaOH soaked filter paper was applied on the central cornea for 1 min and the cornea was washed with 1 mL of PBS. No intervention was applied to the left corneas. From the day of alkali burn, 15 mice were grouped (3 groups and 5 mice in each group) and treated with topical application of phosphate buffered saline (PBS), 100 μM or 1000 μM NaNO_2_ mixed with PBS, one drop, every 6 h for 13 days. Ocular surface pictures of the right eyes were taken at 0 and 14 days after alkali burn. Corneal opacity at day 14 was graded as 0: no opacity, 1: mild opacity but easy visualization of iris vessels, 2: moderate opacity with significant obscuring of iris vessels visualization, 3: severe opacity with no iris vessels visualization. At day 14, mice were sacrificed and the cornea buttons were harvested for the histologic examination.

### Statistical analysis

Data are presented as mean ± standard error, and statistical significance was determined using one-way analyses of variance (ANOVA) and Dunnett’s multiple comparison tests at cGMP assay or western blot analysis and two-way ANOVA followed by the Bonferroni multiple comparison test also carried out at the NO detection analysis. In this study, p < 0.05 was regarded as significant, and calculations were completed with GraphPad Prism v5.01 (GraphPad Software, Inc., La Jolla, CA, USA).

## Supplementary Information

Supplementary Information.

## Data Availability

The datasets generated during and/or analysed during the current study are available from the corresponding author on reasonable request.

## Abbreviations

NO: Nitric oxide
cPTIO: 2-4-Carboxyphenyl-4,4,5,5-tetramethylimidazoline-1-oxyl-3-oxide
cGMP: Cyclic guanosine monophosphate
αSMA: Alpha smooth muscle actin
TGFβ1: Transforming growth factor beta 1
mTOR: Mammalian target of rapamycin
