# Supplementary material for: Nitric oxide attenuated transforming growth factor-β induced myofibroblast differentiation of human keratocytes
Source: Sci Rep. 2021 Apr 14;11:8183. doi: 10.1038/s41598-021-87791-x (PMC8046755; doi:10.1038/s41598-021-87791-x)
Supplement: Supplementary file 1 — Supplementary Information. [file 41598_2021_87791_MOESM1_ESM.docx]

Nitric Oxide Attenuated Transforming Growth Factor-β induced Myofibroblast Differentiation of Human Keratocytes

Joo-Hee **Park** PhD^1^, Bora **Yim** MS^2^, Martha **Kim** MD^2^, Choul Yong **Park** MD, PhD^2^

1. Department of Ophthalmology, Dongguk University, Ilsan Hospital, Goyang, South Korea
2. Department of Biochemistry, Dongguk University, College of Medicine, Gyeongju, South Korea

Word count:

The authors have no proprietary interest in the materials presented herein.

*Correspondence to:

Choul Yong Park MD, PhD

Department of Ophthalmology

Dongguk University, Ilsan Hospital

814, Siksadong, Ilsan-dong-gu, Goyang

Gyunggido, South Korea, 410-773

Tel: 82-31-961-7395

Fax: 82-31-961-7977

Email: [oph0112@gmail.com](mailto:oph0112@gmail.com)

**Supplement Figures**

**Supplement Figure 1.** The expression levels of oncogene activation related proteins, p53 and p21 in TGFβ1 (10 ng/mL) stimulated keratocytes and modulation effect by two NO donors, DETA NONOates (10 or 100 μM) and sodium nitrite (100 or 1000 μM). **A**ddition of DETA NONOate or sodium nitrite showed little effect on p53 or p21 expressions in keratocytes. Statistical significance was determined using one-way ANOVA followed by the Bonferroni multiple comparison test *#* significant difference compared to TGFβ1 treated group (# P<0.05).


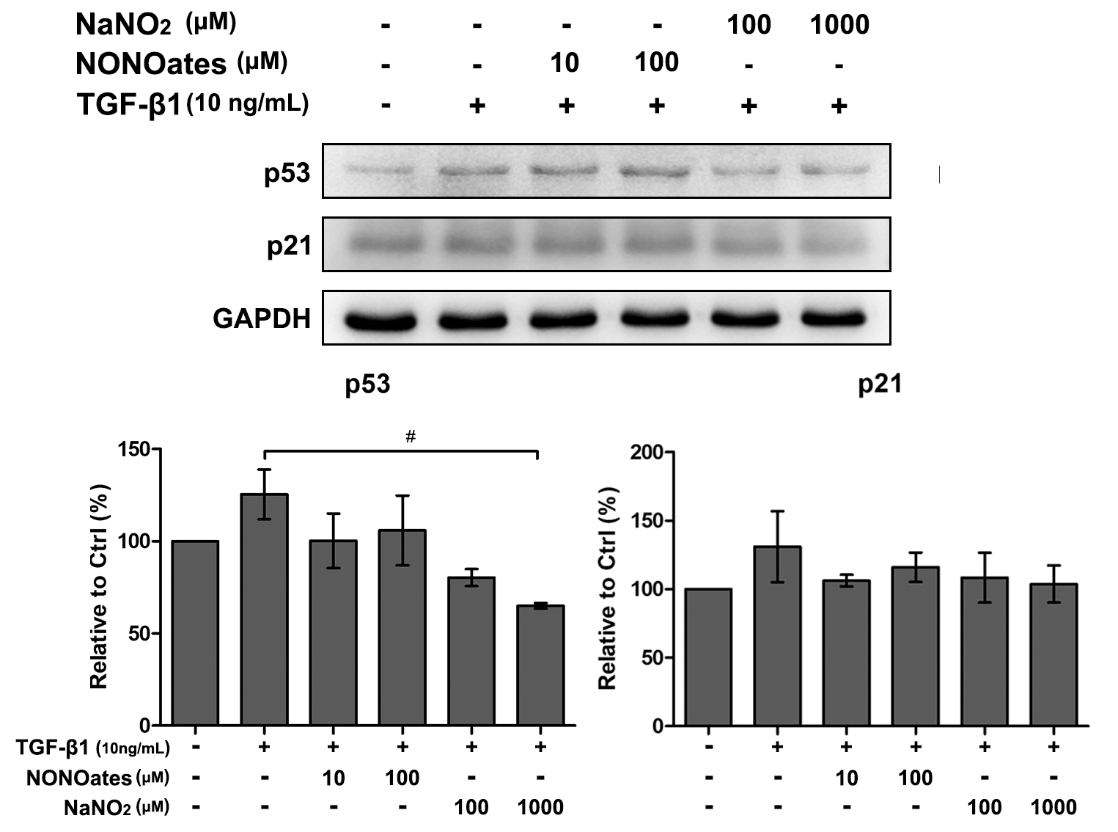


**Supplement Figure 2.** The effect of No donors, DETA NONOates and sodium nitrite on the TGFβ1 stimulated keratocytes. Keratocytes were stimulated by TGFβ1 (Sigma-Aldrich) (10 ng/ml) for 24 hr. For treatment of NO donors, 10 and 100 μM of DETA NONOates (catalog number ALX-430-014, Enzo Life Science, Lausen, Switzerland) or 100 and 1000 μM of sodium nitrite were added to the culture media after 24 h of TGFβ1 stimulation. The expression level of αSMA was evaluated by Western blot. Expression α-SMA in TGFβ1 stimulated keratocytes significantly decreased with the addition of DETA NONOates or sodium nitrite in the culture media. Statistical significance was determined using one-way ANOVA followed by the Bonferroni multiple comparison test and the significant difference compared to no treated control (*P<0.05, ***P<0.001); # significant difference compared to TGFβ1 treated group (## P<0.01)


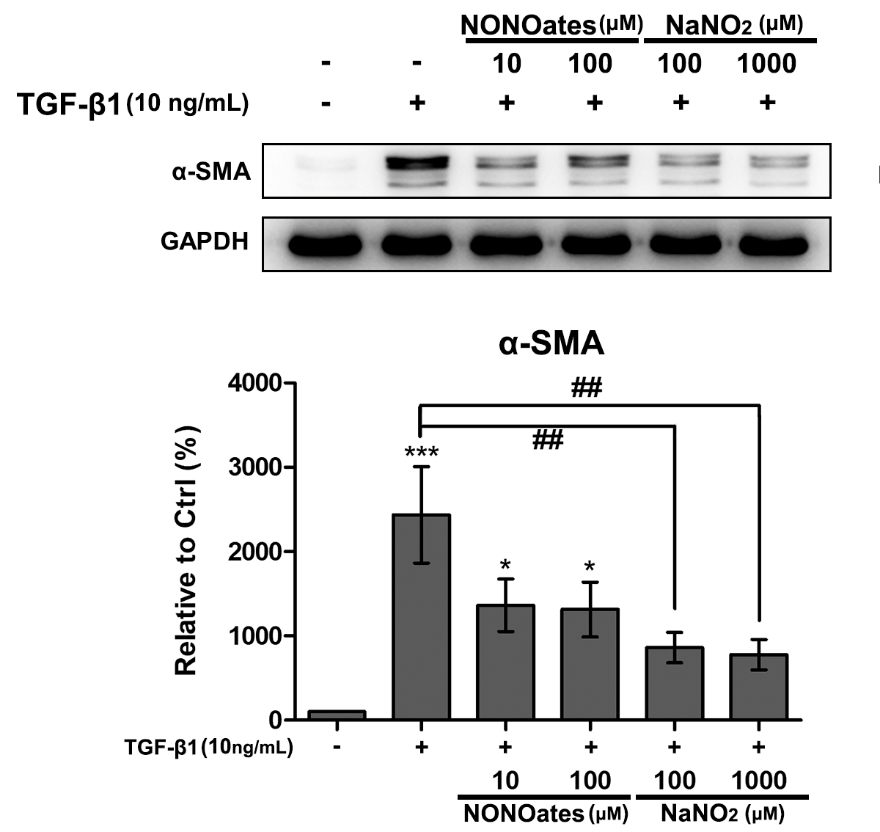


**Supplement Figure 3**. Detection of total S-nitrosothiol in TGF beta stimulated keratocytes with and without sodium nitrite. S-nitrosothiol increased does dependently after addition of sodium nitrite in the culture media for 24 hr in keratocytes. ***P<0.001, ^###^P<0.001


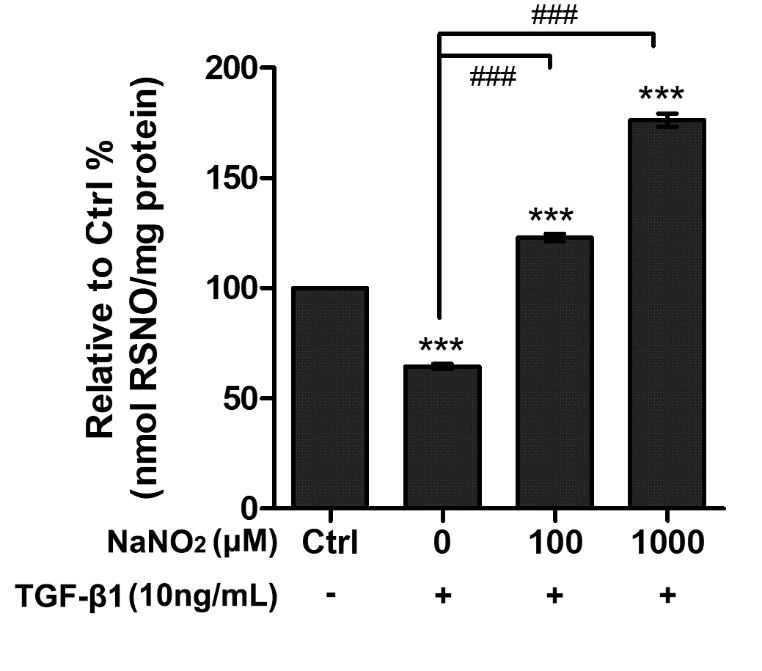
.

**Supplementary Information**

Full length gel of electrophoresis

Figure 3


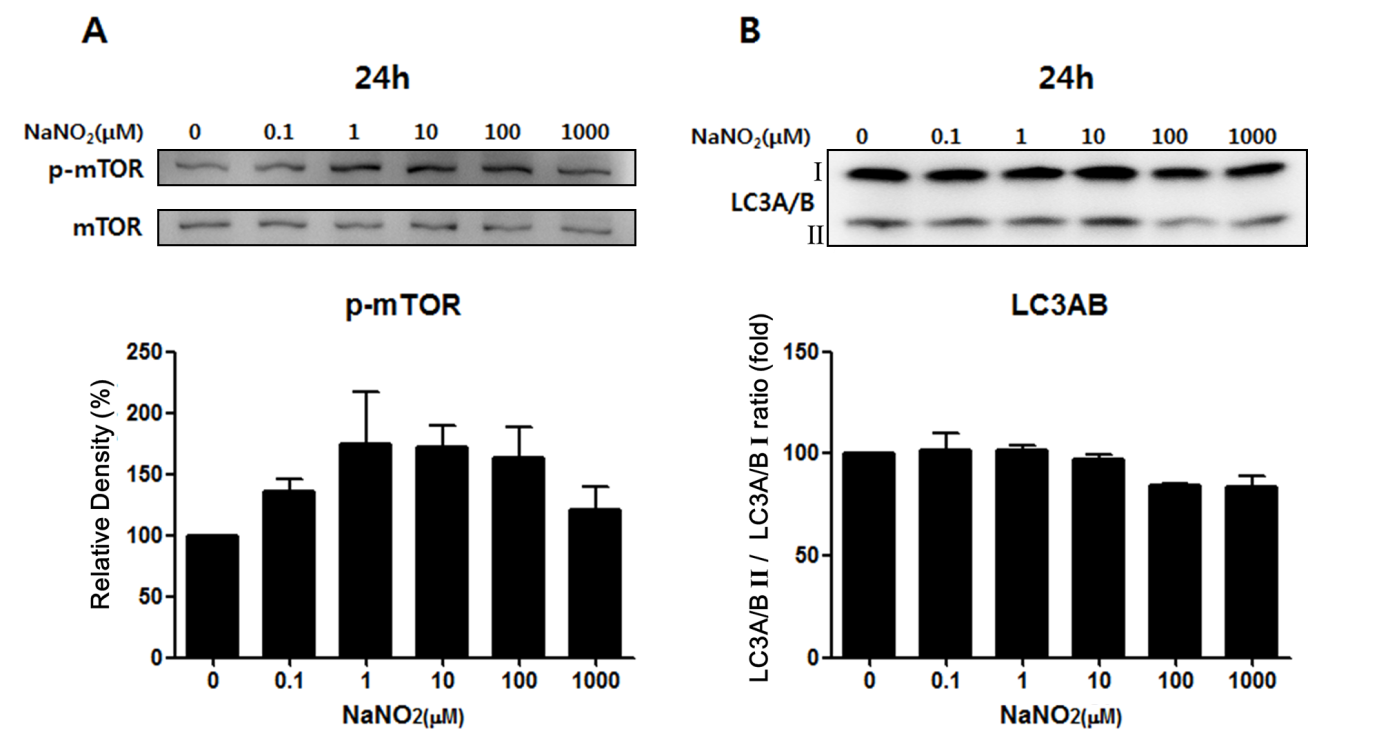


pmTOR (289 kDa)





mTOR (289 kDa)





LC3A/B (14/16 kDa)





Figure 4


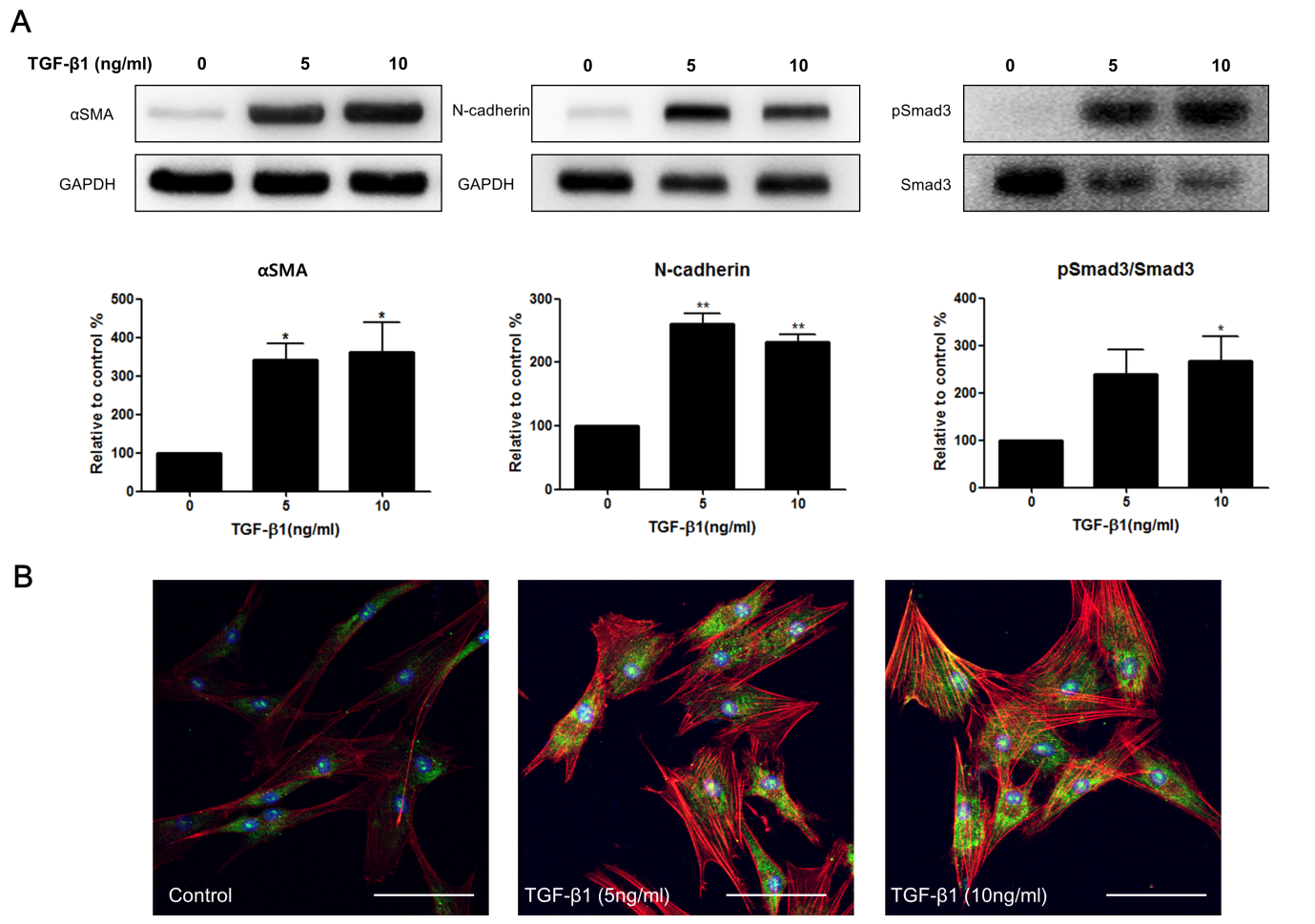


α-SMA (42 ~ 45 kDa)





GAPDH (36 kDa)







N-Cadherin (127 kDa)




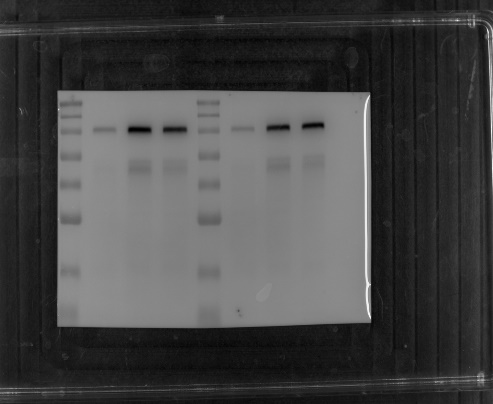


GAPDH (36 kDa)







pSmad3 (48 kDa)







Smad3 (48 kDa)





Figure 6.


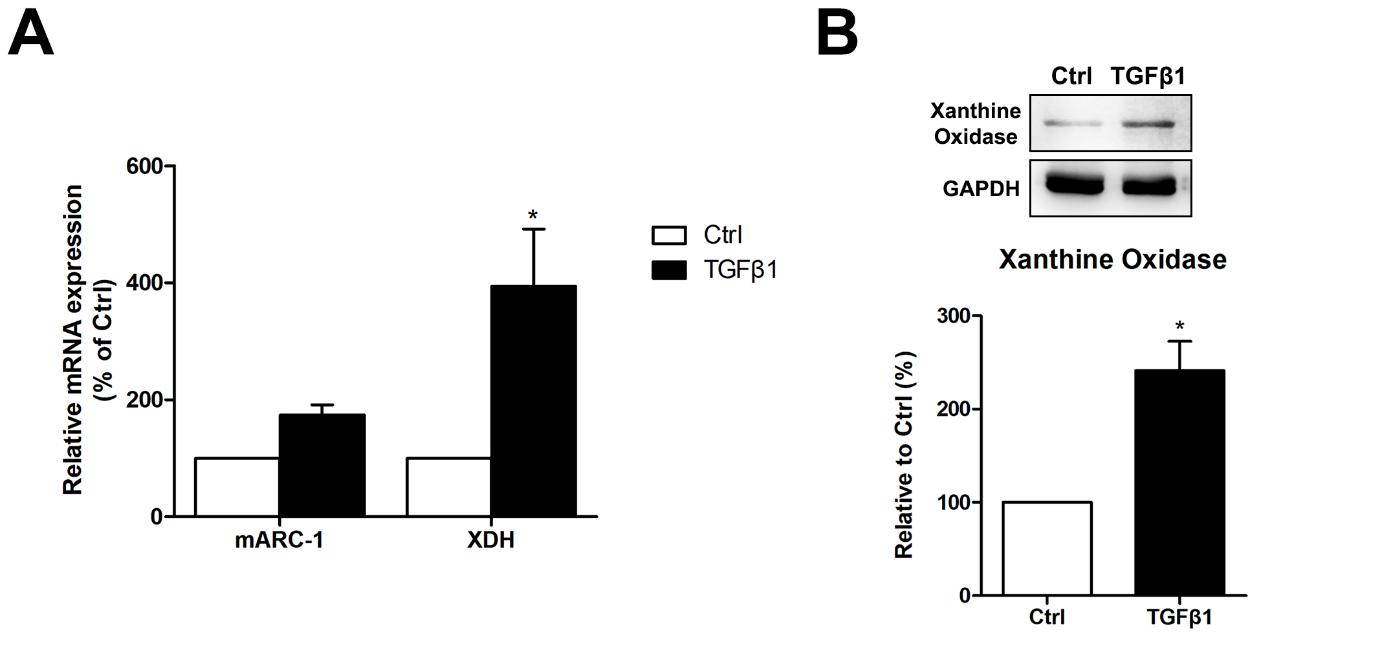


Xanthine oxidase (150 kDa)

***
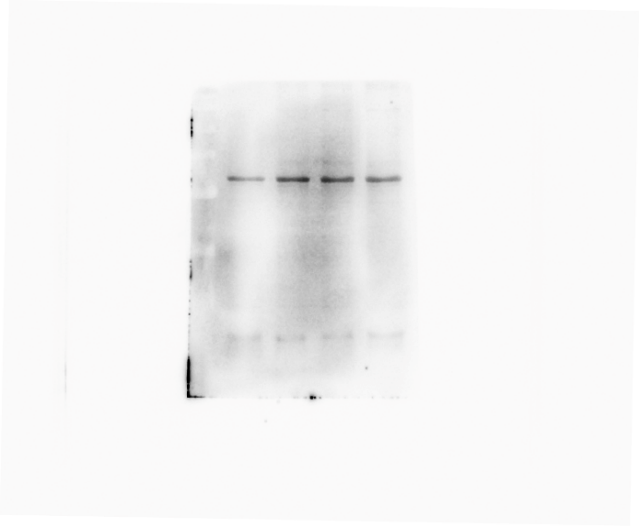
***

GAPDH (36 kDa)

*
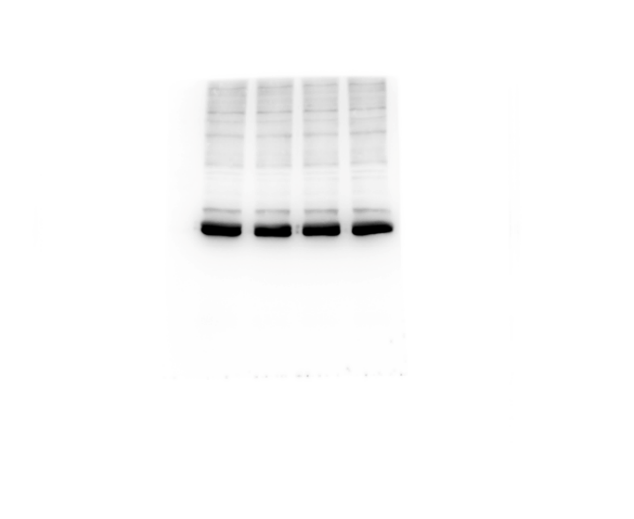
*

Figure 7.


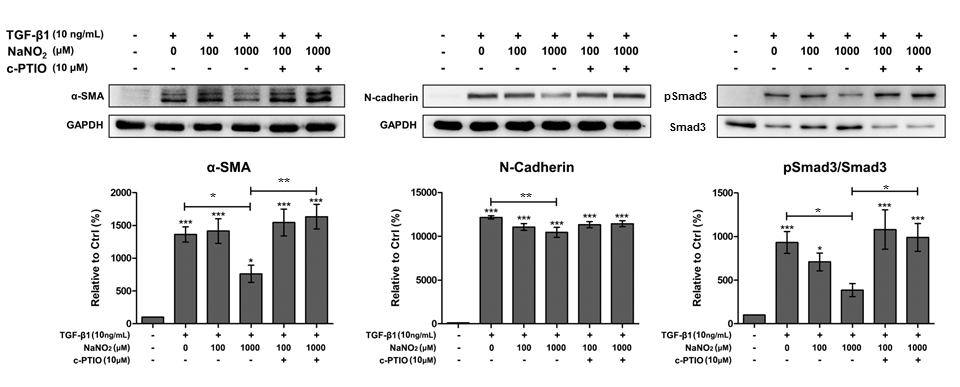


α-SMA (42 ~ 45 kDa)




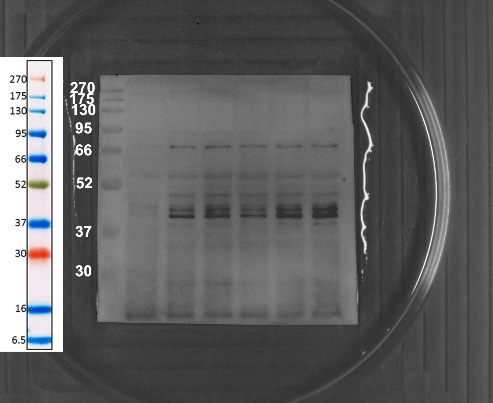


GAPDH (36 kDa)




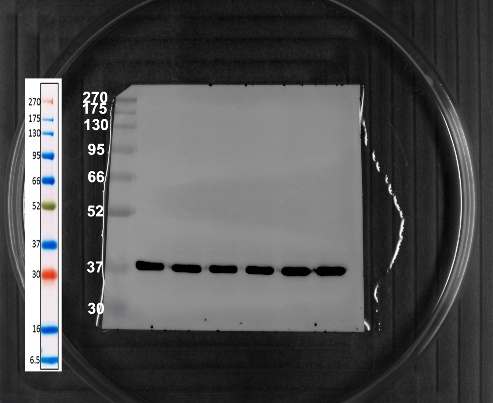


N-Cadherin (127 kDa)




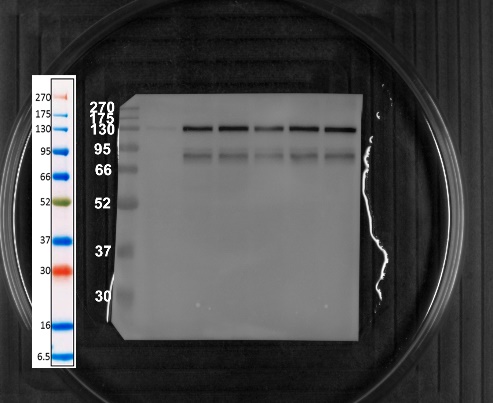


GAPDH (36 kDa)


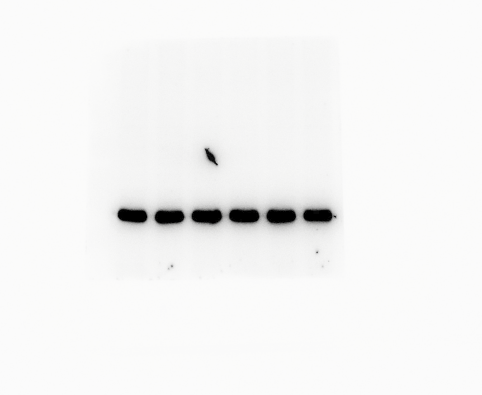

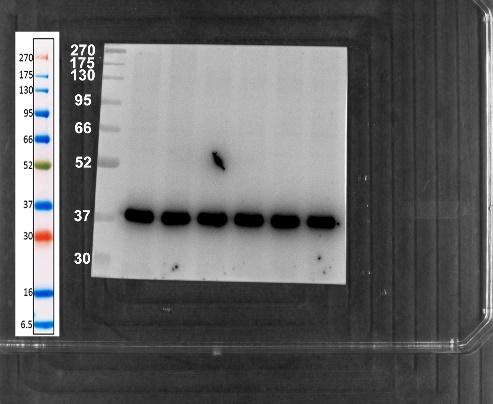


pSmad3 (48 kDa)




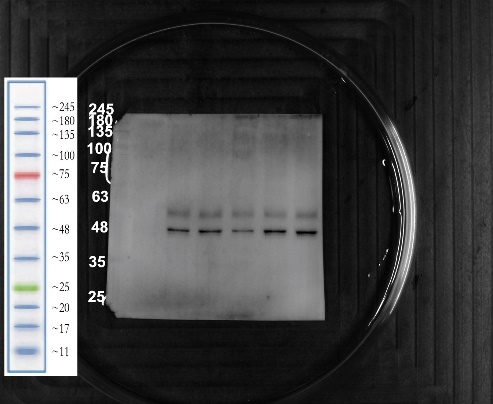


Smad3 (48 kDa)




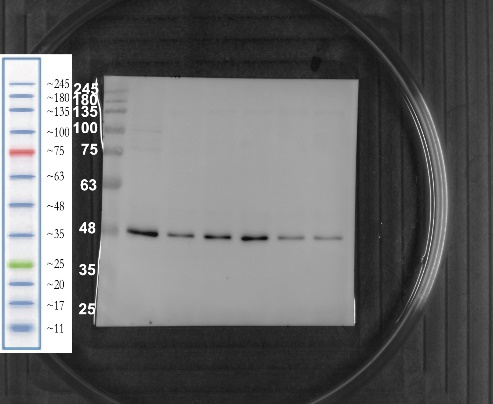


**Supplement Figure 1.**


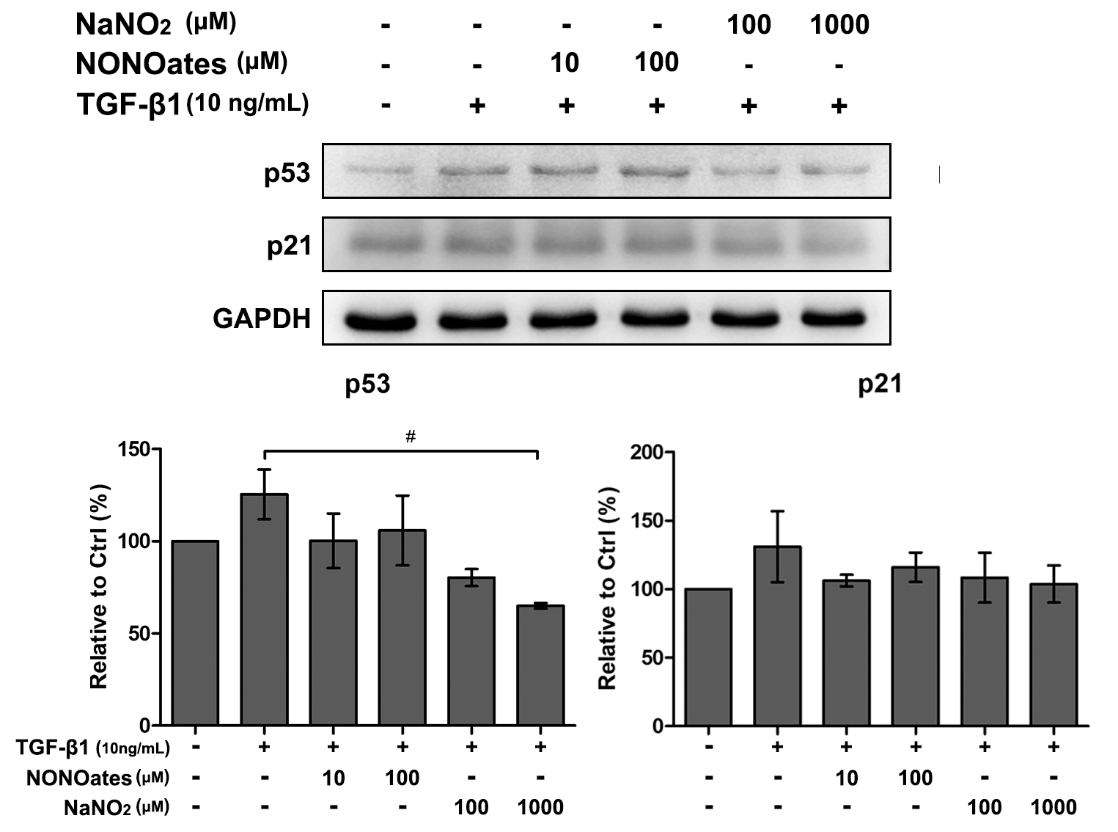


p53




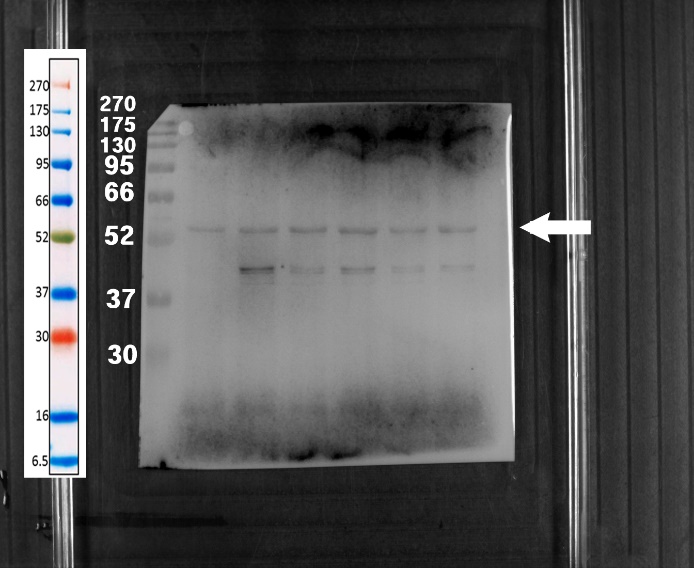


p21 GAPDH
